# The Role of Serological Markers in the Prediction of Disease Course and Response to Therapy in Inflammatory Bowel Disease

**DOI:** 10.7759/cureus.48442

**Published:** 2023-11-07

**Authors:** Maiss Reghefaoui, Tariladei S Peresuodei, Michell S Saavedra Palacios, Abhishek Gill, Chijioke Orji, Tiba Reghefaoui, Jihan Mostafa

**Affiliations:** 1 Internal Medicine, California Institute of Behavioral Neurosciences & Psychology, Fairfield, USA; 2 Medicine, University of Cuenca, Cuenca, ECU; 3 Orthopedics, California Institute of Behavioral Neurosciences & Psychology, Fairfield, USA; 4 Psychiatry and Psychotherapy, California Institute of Behavioral Neurosciences & Psychology, Fairfield, USA

**Keywords:** inflammatory bowel disease, therapy, response, disease course, serological markers, role

## Abstract

Inflammatory bowel disease (IBD) is a common gastrointestinal tract disease and can be divided into two major groups: ulcerative colitis (UC) and Crohn's disease (CD). These two entities can be diagnosed from a combination of invasive and non-invasive tests as well as a thorough history and physical examination. However, invasive tests are preferred for a definitive diagnosis since the two entities have characteristic features of colonoscopy and biopsy. In this review, the following will be discussed: how non-invasive tests could help detect the presence of IBD, how markers help monitor disease progression, and how the disease responds to treatment. Some of the common markers that are discussed in detail include perinuclear antineutrophil cytoplasmatic antibodies (p-ANCA), anti-*Saccharomyces cerevisiae* antibodies (ASCA), C-reactive protein (CRP), erythrocyte sedimentation rate (ESR), calprotectin, lactoferrin, lipocalin-2 (LCN2), and several other novel markers that are based on bacterial antigens. The best non-invasive tests available for detecting the presence of IBD are serological and fecal markers. Detecting these markers has helped doctors significantly by bringing to their attention the possibility of the presence of IBD. The serological testing can also help distinguish the two forms of IBD since a different combination of markers is elevated in UC and CD. In addition, the symptoms of IBD are non-specific and usually overlap with other gastrointestinal tract disorders, so by finding these serological markers, doctors can proceed with further invasive testing that would give them a definitive diagnosis. That way, invasive testing, such as colonoscopy with biopsy, can be avoided in patients with no suspicion of IBD. The common markers used in the clinical setting to point out the presence of IBD are discussed in detail in this review. Recently, more specific markers derived from bacterial antigens are also used, and their role is discussed, too.

## Introduction and background

Various reasons, including infection, toxins, radiation, autoimmune disorders, and ischemia, can cause gastrointestinal inflammation. If none of the mentioned causes are present, a category of idiopathic disorders known as chronic inflammatory bowel diseases (IBD) emerges [1]. Crohn's disease (CD) and ulcerative colitis (UC) are two of the most common and well-known types of IBD [2]. Over the last few years, the prevalence of IBD has increased worldwide. It had long been seen to be a disease of the West; however, data from the last decade show an increase in incidence in Asia, mainly China and India [3]. Recent studies have shown that IBD affects around 0.5-1.0 percent of the Western population [4]. To be precise, CD affects around one in every 650 individuals in the UK, while UC affects approximately one in every 420 individuals [5].

Usually, the disease first appears between adolescence and the third decade of a person's life [6]. In the past, non-biological therapies were the only available therapy that alleviated symptoms but did not modify the disease's course. Nowadays, however, novel treatments have been proposed as a result of breakthroughs in the knowledge of the pathogenic processes involved in IBD, with the most significant development being the introduction of biological therapies [7]. This advancement has helped a lot; however, the pathogenesis of IBD is complex and remains unclear to this date [8]. So far, what is known about the cause of IBD is that both genetic and environmental variables play a major role in its development [9]. Some of the common factors that affect IBD by increasing or decreasing its incidence are shown in Table 1 [10].

**Table 1:** Common factors that affect IBD HRT - hormone replacement therapy; NSAID - nonsteroidal anti-inflammatory drugs; OCP - oral contraceptive pill

IBD	Risk factors increasing incidence
Ulcerative colitis	Diet (arachidonic acid)
	NSAID
	Stress
	Depression
	HRT
	Smoking
	Appendectomy
Crohn's disease	Diet (animal protein)
	NSAID
	Stress
	Depression
	Smoking
	Appendectomy
	OCP
	Dietary fiber
	Vitamin D

Although there have been recent developments in understanding the causes and mechanisms of IBD, we continue to look for a combination of optimal biomarkers to help us enhance the diagnostic and therapeutic methods of IBD. For this reason, we need an ideal biomarker that is easily accessible, non-invasive, inexpensive, and gives accurate and sensitive data in diagnosing and following up on patients with IBD. Researchers are looking for non-invasive biomarkers that may be utilized to give us these data. Many efforts have resulted in the development of several currently used biomarkers, while on the other hand, many of these biomarkers still have flaws that restrict their use. Unfortunately, there is currently no optimal biomarker for assessing and managing IBD. However, there are a few advantages to the newer biomarkers that have been created in recent years that should be acknowledged [11]. We will go through the major biomarkers that have been developed in the past as well as the most recent ones that have been approved to be used to assess the disease course and response to treatment. We will also discuss in detail the major differences between the two forms of IBD and how these markers can help distinguish them.

## Review

The differences and similarities between the two types of IBD

Ulcerative colitis is the first form of IBD that we will discuss in this section. It is an inflammation of only the upper layers of the colon. These inflammatory areas are characteristically widespread and continuous [12]. Additionally, as the name suggests, these inflammatory areas can distinctively be found in the colon only, mainly the distal part of it. If UC remains untreated, it can lead to many complications, and the most common are gastrointestinal bleeding and toxic megacolon. In addition, UC also increases the risk of colorectal carcinoma in the long run. The first-line treatment for UC is medical treatment, meaning that we start with oral and/or rectal drugs such as steroids, immunomodulators, or even novel drugs like biological agents. If these treatments fail, then surgery is indicated. The most optimal and potentially curative treatment option is colectomy [13].

Ulcerative colitis only affects the colon, while Crohn's disease usually affects any part of the gastrointestinal tract from the mouth to the anus. The inflammation caused by the disease is usually transmural. This means that they affect all the layers of the colon, and not the superficial layer only [14]. Unlike UC, CD's lesions are not continuous and are commonly referred to as skip lesions [15]. When an endoscopy with biopsy is performed in patients with Crohn's, it is seen that around 20% of these patients have granulomas. Not only that, but the presence of fissures, fistulae, and strictures throughout their GI tract is also a distinguishing finding of the disease [12].

Usually, patients with IBD, regardless of the type, present mainly with diarrhea, abdominal discomfort, GI bleeding, loss of weight, malnutrition, exhaustion, and malaise [16]. Because the symptoms of the two diseases are so similar, distinguishing one from the other can be challenging [17]. For that reason, a detailed history from the patient, thorough physical examination, and colonoscopy with biopsy are necessary to confirm the presence as well as determine the type of IBD [18]. This is also where the role of serological markers comes into play. Certain serological or fecal markers are usually more elevated than usual in patients with IBD, which we will discuss in detail in the next section.

All in all, UC and CD are classical types of IBD. Each of these diseases has distinguishable histologic and endoscopic features. However, they can be very similar in presentation. Thus, a comprehensive examination, laboratory tests, and certain invasive interventions should be performed to distinguish them. To assist with the identification and monitoring of patients with inflammatory bowel disease, specific serological and fecal markers are usually measured. The elevation of these markers could be considered a red flag for the presence of IBD and could also be used to differentiate its two forms.

**Figure 1:**
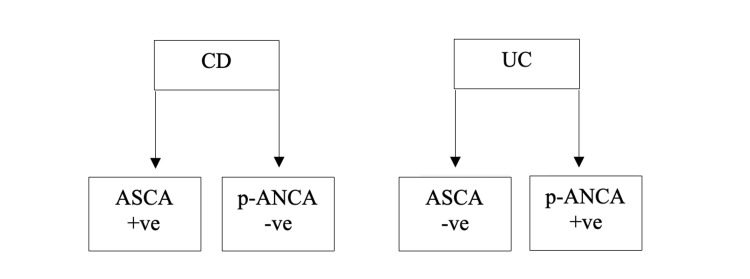
ASCA/p-ANCA antibody combination in Crohn's disease and ulcerative colitis CD - Crohn's disease; UC - ulcerative colitis; ASCA - anti-*Saccharomyces cerevisiae* antibodies; p-ANCA - perinuclear antineutrophil cytoplasmatic antibodies

Common and previously known serological markers and their significance in disease progression and response to therapy:

Various serological markers have been studied in detail for their significance in the disease course and prognosis of patients with IBD. Two of the first markers that were thoroughly studied in the past are anti-*Saccharomyces cerevisiae* antibodies (ASCA) and perinuclear antineutrophil cytoplasmatic antibodies (p-ANCA) [19]. Studies have shown that patients with positive p-ANCA are more likely to have UC, and this elevated level may aid in the identification of the disease [20]. Studies have also shown that ASCAs are more common in CD, where around 40-60% of CD patients were positive for ASCA. Nonetheless, ASCA can also be seen in 4-14% of UC patients. The use of a combination of P-ANCAs and ASCAs to differentiate between the two forms of IBD might be beneficial. It was seen that patients with CD and UC had a different pattern of these antibodies' combinations. Patients with CD were positive for ASCA and negative for p-ANCA, whereas those with UC were negative for ASCA and positive for p-ANCA [21]. Figure 1 summarizes the above.

Other than the discussed antibodies, inflammatory biomarkers in the blood and feces, including C-reactive protein (CRP) and calprotectin, have been studied as predictive indicators [19]. In addition to CRP, another non-specific indicator of inflammation known as erythrocyte sedimentation rate (ESR) is also tested in the blood of individuals with active IBD. Both of these markers are raised in patients with UC and CD, but because ESR and CRP can be raised in a variety of conditions, these markers are not very reliable [11]. Another significant disadvantage of using CRP is that despite active intestinal inflammation, a significant number of patients with UC will not have raised CRP levels.

Measuring the level of the above markers is essential to determine the response to therapy and disease progression in IBD patients. An effective treatment option for severe refractory IBD is the use of anti-tumor necrosis factor (anti-TNF) antibodies. The most common example is infliximab (IFX). Unfortunately, up to 30% of individuals do not benefit from this medication. Studies were conducted to determine if ASCA or p-ANCA might be used to identify IBD patients who would benefit from anti-TNF medication. It was concluded that CD patients with a refractory illness who had the p-ANCA+/ASCA- combination had decreased success rates to benefit from this treatment [22]. Moreover, greater levels of p-ANCA point to a probable link between these antibodies and the resistance of UC patients to this treatment [23]. Additionally, measuring CRP level is a highly reliable way of determining the remission of the disease as well as treatment effectiveness [2]. A study was conducted to measure the level of CRP two weeks after the administration of IFX. Notably, the responders' CRP levels were considerably lower at week two. Therefore, after starting IFX treatment, CRP levels seemed to be a clinically useful marker of treatment responsiveness [24].

Calprotectin is a protein that belongs to the S100 protein family [25] and is present in inflammatory cells' cytoplasm. At room temperature, the protein is stable in feces samples for up to seven days, and a single sample of less than 5g is enough for a valid assay. Increased calprotectin levels in the feces might be used as a screening approach for patients with IBD. It is a simple, non-invasive, and inexpensive screening test that could be used to obtain a presumptive diagnosis of IBD [26]. The only problem limiting its use is the absence of adequate cut-off levels and lack of accuracy in non-colonic illnesses [19]. Nevertheless, increased fecal calprotectin levels can be used as a screening method to identify people who are most likely to have inflammatory bowel illness and indicate the need for an endoscopy as soon as possible. Not only that, but it was also depicted that in both adults and children with suspected IBD, measuring fecal calprotectin initially in a screening test lowers the frequency of endoscopies with negative findings [26].

Another highly specific and sensitive indicator of intestinal inflammation is lactoferrin. It is a glycoprotein that correlates with neutrophil activity. It is a marker that can be used for determining the severity of IBD activity. Measuring fecal levels of lactoferrin gives us a reliable way to help distinguish between IBD and other non-inflammatory bowel diseases. Clinically, a high fecal lactoferrin level suggests the presence of an active IBD. On the other hand, low levels are seen in inactive inflammatory bowel diseases (which include UC and CD) and irritable bowel syndrome. So, this is a great way to distinguish active IBD from these diseases [27].

In 2009, a study was conducted to establish the relevance of fecal calprotectin (FC) and lactoferrin in the prediction of disease relapse. In both CD and UC patients, fecal calprotectin and lactoferrin measurements may be effective in predicting an upcoming clinical relapse, particularly in the next three months. The study detected that patients with high calprotectin concentrations, particularly those above 150 microg/g, had a greater possibility of relapse [28]. This is attributed to the fact that the majority of patients with active IBD who started treatment with anti-TNF drugs had normal FC levels following induction therapy. This normal level was associated with prolonged clinical remission [29].

Lipocalin-2 (LCN2) is a chemically stable protein contained in neutrophil granules and released during inflammation. It is also known as neutrophil gelatinase-associated lipocalin (NGAL) or siderocalin [30]. It is produced during oxidative stress and can operate as a bacteriostatic agent by inhibiting siderophore-mediated iron uptake by bacteria. Siderophores are chemicals that are essential for the survival of bacteria. The bacteria themselves release these chemicals to enable them to obtain iron, which is required for their survival and development. LCN2 has been shown to have a protective effect on intestinal inflammation and regulation by inhibiting bacterial growth using this mechanism. Its expression is substantially elevated during oxidative stress, and this upregulation of LCN2 is usually associated with a compensatory defense mechanism against pathogens that were able to overcome the impaired mucosal barrier that is seen in IBD [31]. In colonic samples taken from biopsies from individuals with IBD, high LCN2 expression by gut epithelial cells was detected [30]. Not only that, but LCN2 levels are also high in the serum of individuals with active IBD [32]. So, elevated LCN2 levels can be measured from the serum and biopsies obtained during colonoscopy.

In conclusion, studies show that high p-ANCA levels were seen in patients with UC while high levels of ASCA were noted in CD patients. The combinations of these antibodies varied amongst patients with UC and CD. As opposed to patients with UC, who were negative for ASCA and positive for p-ANCA, those with CD showed positive ASCA and negative p-ANCA results. CRP, ESR, and calprotectin are all elevated biomarkers in IBD; however, the significance of their elevation is unreliable as they are non-specific markers and can be elevated in other inflammatory conditions. Elevated levels of fecal calprotectin could be used as a screening tool for IBD; however, the only problem limiting its use is the absence of adequate cut-off levels and lack of accuracy in non-colonic illnesses. Lactoferrin is a highly specific and sensitive indicator of intestinal inflammation, and a high fecal lactoferrin level suggests the presence of an active IBD. Measuring fecal calprotectin and lactoferrin may be useful in predicting an upcoming clinical relapse in patients with CD and UC, particularly in the next three months. LCN2 is a protein contained in neutrophil granules and released during inflammation. Biopsies from individuals with IBD showed a high LCN2 expression, and its levels are also high in the serum of individuals with active IBD. 

New, more specific serological markers and their significance in disease progression and response to therapy

With time, researchers started looking for far more sensitive and specific markers, and luckily, specific antibodies targeting bacterial antigens have been discovered [19]. Evidence from previous studies proved the presence of immune dysfunction in response to foreign antigens in the intestine that contributed to the pathogenesis of IBD [33]. These antigens are originating from microbes and have been known to elicit antibody responses. One of the common antigens that leads to such responses is the outer membrane porin C (OmpC) of *Escherichia coli* (*E. coli*) bacteria [34]. Studies have shown that OmpC was positive in 55% of CD patients and around 5-10% of UC patients [35]. Other studies have shown that elevated levels were linked to more severe CD symptoms [36]. 

Another antibody against a bacterial antigen that was discovered is the anti-I2 antibody. I2 is a new gene that was recently discovered in microbes of the Pseudomonas species, namely *Pseudomonas fluorescens* (*P. fluorescens*). I2 is generated from PfiT, which is a gene that is specifically present in *P. fluorescens*, and it entirely accounts for the microbe's T-cell superantigen activity. It was proven, through studies, that PfiT gene has been shown to stimulate CD4+ T cells, making I2 a superantigen. Many microbes, including this one, use these superantigens to destroy tissues by activating the host T-cell immune response. Evidently, these bacteria are highly destructive because of the presence of these genes and virulence factors. In summary, the discovery of I2, which is a new microbial-derived gene sequence encoding a T-cell superantigen, was linked with CD as *P. fluorescens* were present in fresh fecal samples collected from CD patients [37]. It was shown that anti-I2 was positive in 55% of CD cases and only 10% of UC cases, making it more common in CD [35].

The next microbial antibodies that will be discussed are anti-laminaribioside carbohydrate antibody (ALCA), anti-chitobioside carbohydrate antibody (ACCA), and anti-mannobioside carbohydrate antibody (AMCA) [36]. These antibodies are new serologic biomarkers that may aid in diagnosing and categorizing Crohn's disease patients [38]. All three antibodies primarily target the cell wall components of various microbes, such as yeast, fungi, and bacteria. Although the positive rates of ALCA and ACCA are 20-40% in CD and only 10% in UC, a combination of these novel antibodies with ASCA is predicted to increase CD diagnostic value [35]. The diagnosis of CD was proposed with a sensitivity of 77.4% and specificity of 90.6% in patients with IBD positive for antibodies against laminaribioside, chitobioside, or ASCA. The specificity was enhanced to 99.1% when at least two of these antibodies were used and found to be positive [38].

Additionally, antibodies to laminaribioside and chitobioside were the most effective in distinguishing Crohn's disease from ulcerative colitis [38]. ALCA and ACCA, when added to ASCA and p-ANCA, enhanced CD and UC differentiation. ALCA added to ASCA and p-ANCA distinguished CD and UC in the colon. Early onset of CD, perianal illness, and the requirement for surgery were all linked to an increased number of positive antibodies. Not only that, but ALCA and ACCA are also both linked to a more aggressive CD phenotype [39].

Proof has shown that the degree of immune response to various microbial antigens is directly related to the severity of the disease in a given patient; meaning, the greater the immune response, the more severe the disease course and vice versa. Hence, patients who tested positive for I2, OmpC, and ASCA were more likely to have complications like strictures, perforations, and surgery than those who did not. Around 72% of those patients with positive microbial antigens had complications and required surgery [21]. Table 2 shows the incidence of microbial antibodies in both CD and UC.

**Table 2:** Incidence of microbial antibodies in CD and UC OmpC - outer membrane porin C; ALCA - anti-laminaribioside carbohydrate antibody; ACCA - anti-chitobioside carbohydrate antibody

Microbial antibody	CD	UC
OmpC	55%	5-10%
Anti-I2	55%	10%
ALCA and ACCA	20-40%	10%

To summarize, there are now more specific and sensitive markers that target bacterial antigens. These include microbial antigens, which are known to trigger antibody responses. OmpC is present in *E. coli* bacteria and is one of the antigens that trigger these reactions. Research findings indicate that OmpC was positive in over half of CD patients. In addition, higher levels were associated with more severe CD symptoms. Another antibody is the anti-I2, which was more commonly found in cases of CD, with a prevalence of 55% in CD cases and 10% in cases of UC. Other new microbial antibodies are ALCA, ACCA, and AMCA, which aid in diagnosing and categorizing IBD patients. When combined with ASCA and p-ANCA, ALCA and ACCA improved the differentiation of CD and UC.

## Conclusions

IBD is a common gastrointestinal disease with two primary forms: UC and CD. The symptoms of both types are non-specific and overlap, making it hard to distinguish the two forms from each other and other gastrointestinal disorders. For that reason, various markers have been identified to help in the initial recognition of the disease; these markers include p-ANCA, ASCA, CRP, ESR, calprotectin, lactoferrin, LCN2, and many other novel markers that are based on bacterial antigens.

Since testing for these markers is a non-invasive procedure, for many years, all these markers have been preferred to be used by doctors initially if they suspect the presence of IBD, and then proceed with further invasive testing, such as colonoscopy if these markers are present. In addition, these markers have also been used to follow patients up to see their response to the treatment and see how the disease is progressing. This review summarizes the significant markers and how they aid in clinical practice in detecting the disease and monitoring its progression.
